# The CD85j^+^ NK Cell Subset Potently Controls HIV-1 Replication in Autologous Dendritic Cells

**DOI:** 10.1371/journal.pone.0001975

**Published:** 2008-04-09

**Authors:** Daniel Scott-Algara, Vincent Arnold, Céline Didier, Tarek Kattan, Gianluca Pirozzi, Françoise Barré-Sinoussi, Gianfranco Pancino

**Affiliations:** Unité de Régulation des Infections Rétrovirales, Institut Pasteur Paris France; University of California San Francisco, United States of America

## Abstract

Natural killer (NK) cells and dendritic cells (DC) are thought to play critical roles in the first phases of HIV infection. In this study, we examined changes in the NK cell repertoire and functions occurring in response to early interaction with HIV-infected DC, using an autologous *in vitro* NK/DC coculture system. We show that NK cell interaction with HIV-1-infected autologous monocyte-derived DC (MDDC) modulates NK receptor expression. In particular, expression of the CD85j receptor on NK cells was strongly down-regulated upon coculture with HIV-1-infected MDDC. We demonstrate that CD85j^+^ NK cells exert potent control of HIV-1 replication in single-round and productively HIV-1-infected MDDC, whereas CD85j^−^ NK cells induce a modest and transient decrease of HIV-1 replication. HIV-1 suppression in MDCC by CD85j^+^ NK cells required cell-to-cell contact and did not appear mediated by cytotoxicity or by soluble factors. HIV-1 inhibition was abolished when NK-MDDC interaction through the CD85j receptor was blocked with a recombinant CD85j molecule, whereas inhibition was only slightly counteracted by blocking HLA class I molecules, which are known CD85j ligands. After masking HLA class I molecules with specific antibodies, a fraction of HIV-1 infected MDDC was still strongly stained by a recombinant CD85j protein. These results suggest that CD85j^+^ NK cell inhibition of HIV-1 replication in MDDC is mainly mediated by CD85j interaction with an unknown ligand (distinct from HLA class I molecules) preferentially expressed on HIV-1-infected MDDC.

## Introduction

Natural killer (NK) cells are a key component of innate immune responses to pathogens and tumour cells, through cytokine secretion and lysis of abnormal cells [1]–[3]. NK function is regulated by a balance between activating and inhibitory signals that are generated by an array of NK cell surface receptors (NKR) upon engagement by specific cellular ligands [1], [4]. NK inhibitory receptors specific for MHC class I molecules, by delivering inhibitory signals to NK cells, can prevent unwanted responses to normal cells, that express a complete set of self MHC molecules [1]. Thus, NK cell interaction with normal autologous cells leads to NK cell inactivation. However, dendritic cells (DC) are an exception to this rule, as they are targeted by NK cells despite normal MHC class I expression. It has recently been shown that the cognate interaction between NK cells and DC results in crosstalk [3], [5] thought to be crucial for optimal induction of both innate and adaptive responses. DC can prime and induce proliferation of NK cells, which, once activated, acquire the ability to kill immature DC (iDC), through engagement of the activating receptor NKp30, and may also induce DC maturation [5], [6]. DC infected by pathogens upregulate HLA class I, become resistant to NK-mediated lysis, and express CCR7, thus acquiring the ability to migrate to secondary lymphoid organs where the adaptive immune response takes place [3].

Both NK cells and NK-DC interactions are affected by HIV-1 infection [7]–[11]. A deregulation of NK cell subset distribution and function was already described during acute HIV-1 infection, with elevated NK cell numbers, expansion of CD3^-^CD56^(dim)^ NK cells, and early depletion of CD3^-^CD56^(bright)^CD16^−^ NK cells [12], [13]. In chronic infection viremic patients showed an accumulation of CD3-CD56-CD16+ NK cells [14]. NK cell activity correlates negatively with the level of viral replication and declines rapidly in patients starting highly active antiretroviral therapy [15]. Both NK cell cytoxicity and cytokine production are impaired in HIV-1 viremic patients [16]. Accordingly, the expression of activating and/or inhibitory receptors is altered in these patients [17]. It has also been shown that NK cells from HIV-1-infected patients are defective in their capacity to lyse autologous monocyte-derived iDC[11]. In addition, in vitro interactions between a CD56^-^CD16^+^ subset of natural killer (NK) cells and autologous DC from HIV-1-infected viremic individuals (but not aviremic individuals) are markedly impaired and likely interfere with the development of an effective immune response [9]

However, there is also evidence suggesting a protective role of NK cells in HIV-1 infection Preserved NK cell activity and number correlate with lower plasma viral load and slower progression to the acquired immune deficiency syndrome (AIDS) [15], [18]. The combined expression of the activating KIR3DS1 receptor and the HLA-B Bw4-80I allele has been associated to slower disease progression [19]. Furthermore, KIR3DS1^+^ NK cells can inhibit HIV-1 replication in vitro in autologous HIV-1-infected Bw4-80I CD4^+^ T cells [20]. High *ex vivo* NK cell cytotoxic activity and cytokine production, in association with the expansion of particular NK cell subsets, have been found in HIV-1-exposed but uninfected (EU) Vietnamese intravascular drug users (IDU) [21], [22]. Altogether, these data point to a protective role of NK cells against HIV infection and disease.

NK cell responses are induced very early in HIV infection [12]. The innate immune response against the virus is likely crucial both for the subsequent course of the infection and for the induction of an efficient adaptive response. NK cells encounter DC in sites of infection or inflammation, and their interaction may be modified by HIV-1 infection. Indeed, iDC infection by HIV-1 alters their maturation and their capacity to secrete cytokines [23]. These alterations may, in turn, affect their interaction with NK cells, modulating both NK cell receptor expression and function. Conversely, activated NK cells may exert antiviral activity and protect DC from HIV-1 infection.

To study the impact on NK cell receptor repertoire and functions of DC infection by HIV-1, and to address the role of NK cells in controlling DC infection, we set up an *in vitro* model of NK cell coculture with HIV-infected autologous monocyte-derived DC (MDDC). We found changes in the expression of several NK cell receptors and, remarkably, a potent capacity of a particular NK cell subset, expressing the CD85j receptor, to suppress viral replication in infected MDDC.

## Results

### NK cell interaction with HIV-1-infected MDDC modifies NK cell repertoire expression

**Figure 1:**
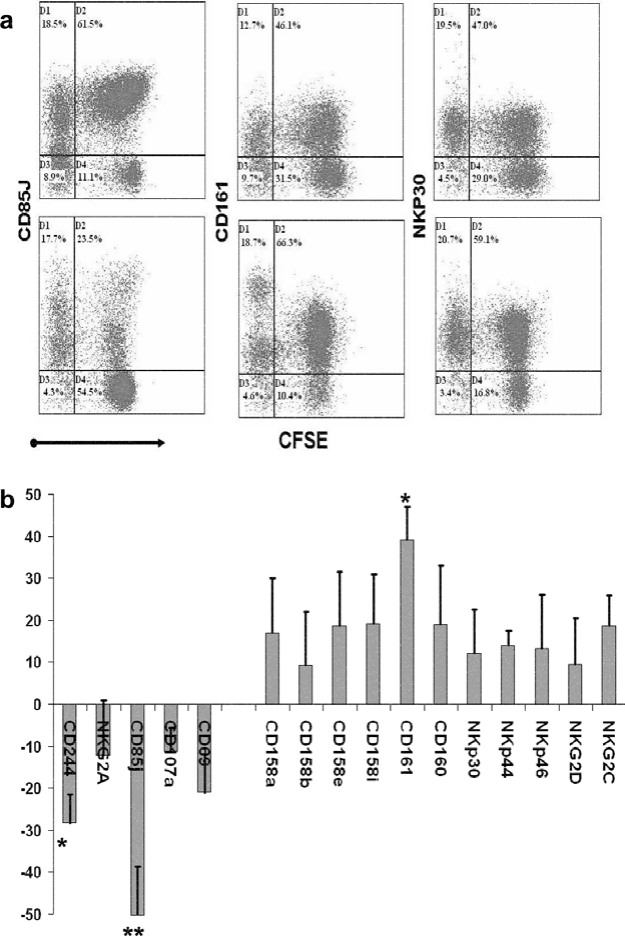
Changes in NK cell receptor expression after coculture with HIV-1-infected MDDC. (a) Dual-label flow cytometric analysis of NK cell proliferation (decrease in CFSE fluorescence) and NKR expression gated on CD16/CD56^+^CD3^−^ cells. NK cells were stained after 5 days of coculture with uninfected (top) or HIV-1-infected (bottom) MDDC. A representative experiment showing the expression of three NK cell receptors differently modulated by HIV-1-infected MDDC, CD85j (down-regulated), CD161 (up-regulated) and NKp30 (not significantly changed). (b) Overall modifications in the percentage of NKR expression on NK cells after coculture with HIV-1-infected MDDC. Results are expressed as the percentage decrease (negative values) or increase (positive values) in NK cells expressing the different receptors after coculture with HIV-infected MDDC in comparison to coculture with uninfected MDDC. Means+SE of 9 independent experiments. * p<0.05, **p<0.01

After coculture with autologous uninfected MDDC we observed NK cell proliferation and up-regulation of all the NK cell receptors analyzed, in agreement with previous reports (data not shown). We then investigated NK cell proliferation and receptor expression after co-culture with HIV-infected MDDC. The percentages of proliferating NK cells, indicated by a decrease in CFSE fluorescence, were not significantly different between cocultures with uninfected MDDC (Fig 1a top panels) and HIV-1-infected MDDC (Fig 1a bottom panels). In contrast, some NK receptors were differently modulated by HIV-1-infected and uninfected MDDC (Fig 1a,b). For example, the expression of CD85j and CD161 on NK cells cocultured with HIV-1-infected MDDC was decreased and increased, respectively, compared to their expression after coculture with uninfected MDDC (Fig. 1a,b), whereas NKp30 was upregulated to similar levels in both conditions. Despite variations among the donors, a strong decrease in CD85j receptor expression on NK cells was consistently observed after coculture with HIV-infected MDDC (Fig 1b). We also observed a significant increase in CD161 expression and a decrease in CD244 expression (Fig 1b).

### CD85j^+^ NK cells inhibit HIV-1 replication in infected MDDC

**Figure 2:**
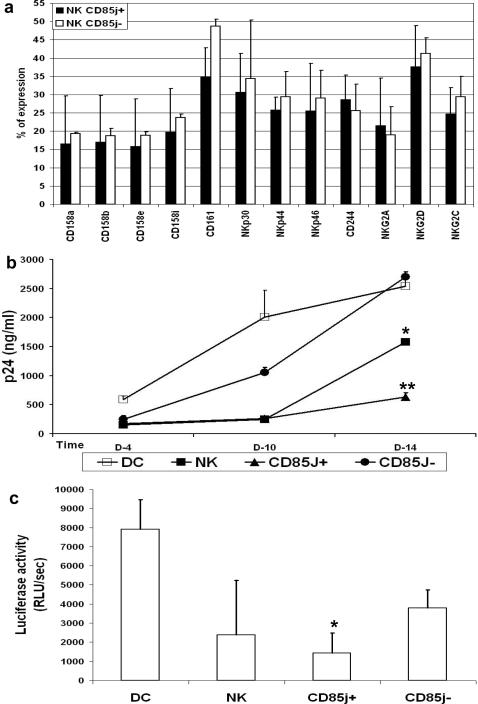
Suppression of HIV-1 replication in infected MDDC by the CD85j^+^ NK cell subset. (a) NKR expression on purified CD85j^+^ and CD85j^−^ NN cell subsets. Results are expressed as percentage of receptor expression in the given subset+SE. (b) MDDC were infected with HIV-1_Bal_ and then cultured alone (DC) or incubated with total NK cells (NK) or with the CD85j^+^ or CD85j^−^ NK cell subsets. HIV-1 replication was measured by p24 production at the indicated time points. Mean+SE of p24 levels in 8 independent experiments using cells from different donors. p24 levels in NK/MDDC cocultures were compared to those in MDDC cultures * p<0.02, **p<0.001. (b) MDDC were one-round infected with HIV-1/BaL pseudotypes and then cultured alone (DC) or incubated with total NK cells (NK) or with the CD85j^+^ or CD85j^−^ NK cell subsets. Ninety-six hours later, cells were lysed and luciferase activities were measured. Results are expressed as means±SE of luciferase activity of 4 independent experiments. CD85j^+^ NK cells induced higher levels of inhibition than CD85j^−^ NK cells. * p<0.05

As CD85j was the NK receptor whose expression was by far the most strongly altered in coculture with HIV-1-infected MDDC, we investigated the impact of the CD85j^+^ NK cell subset on HIV-1 replication in MDDC. We isolated CD85j-enriched (CD85j^+^) and CD85-depleted (CD85j^−^) NK cells and analyzed NKR expression on the two subsets. CD85j^+^ and CD85j^–^ NK cells did not differ in their expression of other NKR (Fig. 2a). MDDC were infected with HIV-1_Bal_ and incubated 2 hours later with the CD85j^+^ or the CD85j^–^ NK cell subset, in parallel with the total NK cell population. Viral replication, reflected by Gag p24 protein levels in culture supernatants, was strongly decreased in MDDC cocultured with CD85j^+^ NK cells compared to MDDC without NK cells (Fig. 2b). A modest and transient decrease of p24 was observed in MDDC cocultures with CD85j^−^ NK cells. An intermediate level of HIV-1 inhibition was observed in MDDC cocultured with total NK cells that comprise both CD85j^+^ and CD85j^−^ NK cells (Fig. 2b). Suppression of HIV-1 replication in MDDC by CD85j^+^ NK cells was observed in 14 out of 17 donors tested.

To assess whether CD85j^+^ NK cells can affect viral replication already at the first cycle, we used an envelope-defective luciferase reporter HIV-1 virus complemented with the HIV-1 BaL Env in single-round infections. In this system, luciferase expression reflects the expression of early HIV-1 genes, after completion of the first steps of viral replication. Addition of CD85j^+^ NK cells to infected MDDC achieved significantly stronger inhibition of HIV-1 replication, as measured by luciferase activity in cell lysates, than CD85j^−^ NK cells (Fig 2c). These results indicate that CD85j^+^ NK cell-mediated inhibition affects a post-entry step of HIV-1 replication before translation of early viral proteins.

### HIV-1 suppressive capacity of CD85j^+^ NK cell is induced by infected MDDC despite decreased cytotoxic activity

**Figure 3:**
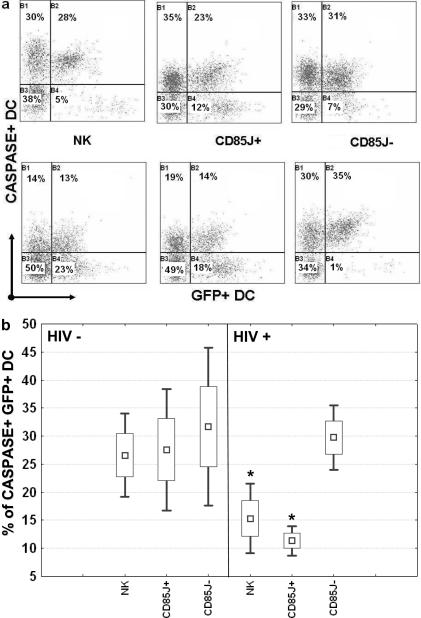
Decrease in CD85j^+^ NK cell cytotoxic activity against DC after co-culture with HIV-1-infected DC. (a) Cytolytic activity of total NK cells (NK), CD85j-enriched NK cells (CD85j+) and CD85j-depleted NK cells (CD85j−) derived from 5-day cocultures with uninfected MDDC (top panels) or HIV-1-infected MDDC (bottom panels) were analyzed for their lysis of HIV-1/GFP infected MDDC. Lysis of target MDDC replicating HIV (GFP+) and not replicating HIV (GFP-) are measured by caspase staining (quadrants B1 and B2, respectively). A representative experiment is shown. (b) Percentages of lysis of HIV-1/GFP+ MDDC target cells by total NK cells or by the CD85j^+^ or CD85j^−^ subset coculture with uninfected MDDC (HIV-, left panel) or with HIV-1-infected MDDC (HIV+, right panel). Results of 4 independent experiments are summarized. Boxes and whisker plots show the percentiles and median distribution of caspase-stained MDDC. Significant differences between NK cells from cocultures with uninfected MDDC and NK cells from cocultures with HIV-1_ Bal_-infected MDDC are indicated, **p<0.01.

**Figure 4:**
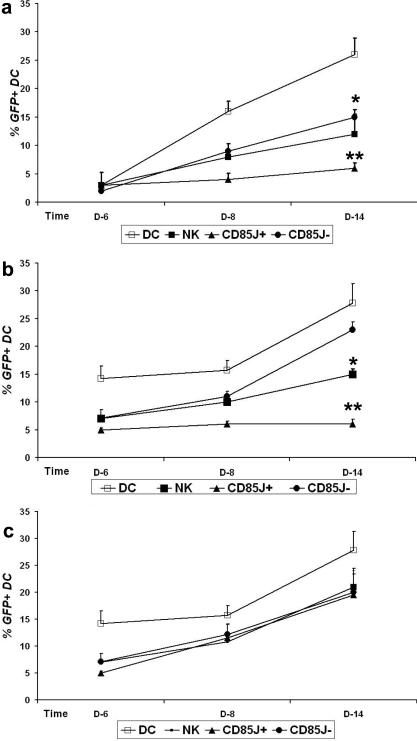
Maintenance of CD85j^+^ NK cell suppression of HIV-1 replication in DC despite the loss of cytotoxic activity after co-culture with HIV-1-infected DC. (a) NK cells (NK) or the CD85j^+^ or CD85j^−^ NK cell subsets were added to HIV-1/GFP-infected MDDC 2 hours after infection. (b, c) NK cells or the CD85j^+^ or CD85j^−^ NK cell subsets derived from 5-day cocultures with HIV-1_Bal_-infected MDDC (b) or uninfected MDDC (c) were added to HIV-1/GFP-infected MDDC. Kinetics of HIV-1/GFP replication are expressed as percentages of MDDC cells expressing GFP (means+SE) at the indicated time points in eight independent experiments (a) or three independent experiments (b,c) Significant differences between HIV-1/GFP-infected MDDC cultures without NK cells (DC) and MDDC/NK cocultures on day 14 are indicated * p<0.05, **p<0.001.

To evaluate whether the inhibition of HIV-1 replication in infected DC by CD85j^+^ NK cells might be related to NK cytolytic activity, we first analyzed the effect of co-culturing NK cells with DC infected or not infected by HIV-1 on NK cytolytic activity. The three NK cell populations showed similar lytic capacities after coculture with uninfected MDDC (Fig. 3a, top panels and Fig. 3b, left panel). In contrast, total NK cells and the CD85j^+^ subset, but not by the CD85j^−^ subset, showed a significant reduction in their lytic activity after coculture with HIV-1-infected MDDC in comparison with coculture with uninfected MDDC (Fig 3a,b). Similar trends were observed for the lysis of GFP^−^ and GFP^+^ MDDC (Fig 3a, B1 and B2 quadrants) suggesting that HIV replication in MDDC does not modify MDDC susceptibility to NK cell lysis. The decrease in the cytolytic capacity of CD85j^+^ NK cells after coculture with HIV-1-infected MDDC suggested that the inhibition of HIV-1 replication in MDDC by this NK cell subset was not mediated by cytoxicity. However, we could not exclude the possibility that CD85j^+^ NK cells might have “exhausted” their cytotoxic activity during coculture with infected MDDC and then lost their suppressive activity. To specifically address this point, we examined whether the anti-HIV-1 activity of CD85j^+^ NK cells was maintained after coculture with HIV-1–infected MDDC, despite the decrease in their cytolytic capacity. Total NK cells and CD85j^−^ NK cells were studied in parallel. After 5 days of coculture with HIV-1_Bal_-infected MDDC, each subset of NK cells was incubated with MDDC previously infected with HIV-1/GFP and the GFP expression in MDDC was monitored. HIV-1/GFP was used in these experiments despite not expressing the Nef protein that affects the surface expression of HLA class I molecules (ligands for CD85j and other NK cell receptors), since the levels of HIV-1 suppression by CD85j NK cells in DC infected with HIV-1/GFP or with the HIV-1_Bal_ strain that express Nef were equivalent (compare Fig 4a with Fig. 2b). The CD85j^+^ NK cell subset was still able to suppress HIV-1 replication, inducing levels of inhibition similar to those achieved when added to HIV-1-infected DC immediately after infection (Fig. 4a,b). The percentage of GFP^+^ MDDC in cocultures with CD85j^+^ NK cells was significantly reduced throughout the study period in both cases, in comparison with HIV-1/GFP-infected MDDC cultured without of NK cells (Fig. 4a,b). As already observed in previous experiments, CD85j^−^ NK cells only partially controlled HIV-1/GFP replication, while total NK cells induced an intermediate level of control (Fig. 4a,b). Interestingly, only a not stastistically significant decrease of GFP^+^ MDDC was induced by both CD85j^+^ and CD85j^−^ NK cells from cocultures with uninfected MDDC (Fig. 4c), despite both subsets maintained cytolytic capacity (see Fig. 3b). These results confirmed that the HIV-suppressive capacity of CD85j^+^ NK cells is induced by HIV-1-infected MDDC, and that it cannot be explained by their cytolytic capacity.

### Control of HIV replication in DC by CD85j^+^ NK cells requires NK-DC contact and does not apparently depend on soluble factors

**Figure 5:**
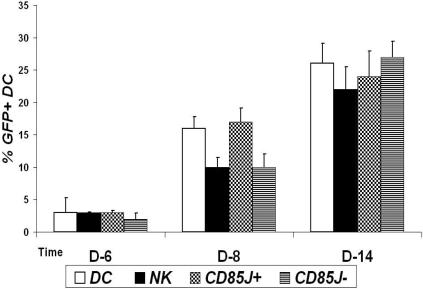
Suppression of HIV-1 replication in DC by CD85j^+^ NK cells requires cell-to-cell contact and does not apparently depend on soluble factors. Total NK cells or the CD85j^+^ or CD85j^−^ subsets were cocultured with HIV-1_Bal_-infected MDDC in the bottom of Transwell wells. HIV/GFP-infected MDDC were added to the inserts and GFP expression was monitored over time. The percentages of NK cells expressing GFP in the inserts placed on the different cell cultures are shown: MDDC cultures without NK cells (DC), with total NK cells (NK), with CD85j^+^ NK cells (CD85j^+^) and with CD85j^−^ NK cells (CD85j^−^). Means+SE of 4 independent experiments. Significant differences between HIV-1/GFP-infected MDDC in transwell on MDDC/NK cocultures and on MDDC alone * p<0.05.

Activated NK cells secrete molecules that can control HIV infection, such as β-chemokines and other inhibitory factors [24]. We then used transwell experiments to determine whether soluble inhibitory factors, such as β-chemokines, secreted by NK cells in coculture with HIV-1-infected MDDC, were involved in the control of HIV infection, especially by the CD85j^+^ subset. Total NK cells and NK cell subsets were incubated with HIV-1_Bal_-infected DC or with uninfected DC (not shown), and HIV-1/GFP-infected DC were then added, as target cells, to transwell inserts that were placed in the NK-DC coculture-containing wells. A transient decrease in the percentage of GFP^+^ MDDC on day 8 was observed in the inserts placed on total NK cells or CD85j^−^ NK cells cocultures with HIV-1-infected MDDC (Fig. 5). This weak and transient inhibition of HIV-1/GFP replication may be due to the secretion of HIV-inhibiting molecules by NK cells upon contact with infected MDDC. In contrast, CD85j^+^ NK cells cocultures with HIV-1_Bal_-infected DC did not affect HIV-1/GFP infection of DC in the inserts (Fig. 5). These results indicate that that the suppression of HIV-1 replication by CD85j^+^ NK cells in MDDC requires NK-MDDC contact but it is unlikely due to soluble factors. Accordingly, intracellular β-chemokine staining did not show differences in the percentages of stained CD85j^+^ NK cells after 5 days of coculture with HIV infected MDDC compared to coculture with normal MDDC (results not shown).

### The suppression of HIV replication in MDDC by CD85j^+^ NK cells requires CD85j receptor interaction with MDDC surface ligands

**Figure 6:**
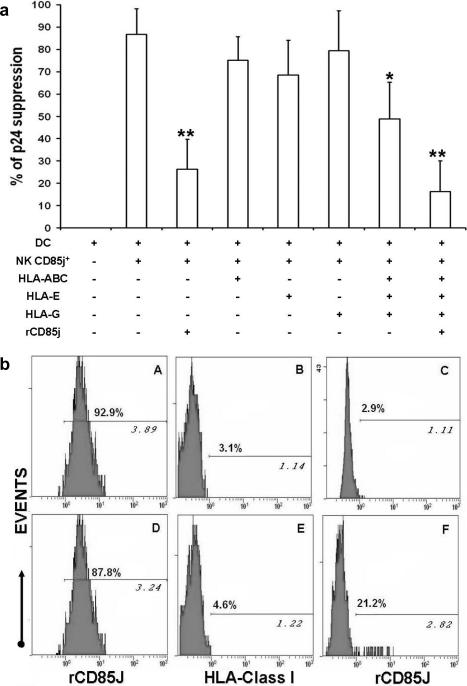
Involvement of NK CD85j receptor interaction with CD85j/ligands, including non-HLA class I ligands, in the control of HIV-1 replication in MDDC. (a) MDDC were infected with HIV_Bal_ and incubated with CD85j^+^ NK cells after blocking CD85j receptor interactions with HLA class I molecules on the MDDC by treatment with Mabs to HLA class I ABC (HLA-ABC), E (HLA-E) or G (HLA-G) or by incubating MDDC with a recombinant CD85j protein (rCD85j). Blocking reagents were also added during coculture. MDDC infection was evaluated by measuring p24 in the supernatants 10 days later. The results are expressed as the percentage suppression of p24 (mean+SE) in comparison with HIV_Bal-_infected MDDC not exposed to NK cells in 8 independent experiments. Significant differences in the levels of p24 suppression after incubation with the three anti-HLA class I Abs, with rCD85j or with rCD8j more the three anti-HLA class I Abs in comparison to CD85j^+^ NK cells cultured with infected DC without Abs are indicated by asterisks: * p<0.05, **p<0.001 (b) Flow cytometric analysis of rCD85j binding to uninfected MDDC (top panels) or to HIV_Bal_-infected MDDC (bottom panels). MDDC staining with rCD85j as revealed by anti-hIgG-PE Abs (A and D). MDDC staining with a mix of anti-HLA Class I-PE mAbs after incubation with rCD85j (B and E), or with rCD85j and anti-hIgG-PE Abs after incubation with a mix of anti-HLA Class I MAbs. A representative experiment of three is shown. Without rCD85j blockade, nearly all MDDC were stained by anti-HLA-PE (not shown). The concentrations of anti-HLA class I Abs used in blocking experiments (10 µg/ml of each anti-HLA class I A,B,C, E and G) totally prevented the detection of HLA class I molecules on MDDC by using labelled anti-HLA class I Abs (not shown). Percentages of positive cells and MFI (*italic*) are shown. Range of positive rCD85j cells was between 8.2 and 21.9.

The transwell experiments indicated that the suppression requires cell-to-cell contact. Furthermore, the down-regulation of CD85j expression observed on NK cells in contact with HIV-1-infected MDDC suggested engagement of the CD85j receptor. We therefore examined the role of the CD85j receptor interaction with its ligands on MDDC. Known CD85j ligands include classical and nonclassical HLA class I molecules. Thus, we used blocking antibodies specific for HLA-ABC, -E or –G to inhibit their interaction with CD85j. Furthermore, to block other potential interactions between CD85j and unknown ligands, we used a recombinant CD85j protein (rCD85j). Incubation of HIV-infected MDDC with CD85j^+^ NK cells diminished p24 production by about 90% compared to infected DC not exposed to NK cells. (Fig. 6a). Whereas antibodies blocking only one type of HLA (ABC, E or G) did not significantly modify CD85j^+^ NK cell-mediated viral suppression, the three anti-HLA Class I blocking Abs together only partially decrease p24 suppression (p<0.05) (Fig. 6a). Notably, incubation with rCD85j strongly reduced viral suppression (p<0.001). Viral suppression was further reduced by simultaneous incubation of HIV-infected MDDC with both the three anti-HLA class I antibodies and rCD85j (Fig. 6a). The removal of HIV-1 inhibition by rCD85j was not attributable to loss of NK cells by cytotoxicity, since NK cell number was comparable in NK-MDDC cocultures with or without rCD85j (data not shown). These results suggest that the control of HIV infection by the CD85j^+^ NK subset requires CD85j interaction with ligands expressed on infected MDDC, which may include, but are not limited to HLA Class I molecules. We then measured CD85j ligand expression on MDDC by flow cytometry. Approximately 90% of MDDC, either uninfected (top panel) or HIV-1-infected (bottom panel), were stained by rCD85j (Fig. 4b, A, D panels). In parallel, we incubated uninfected and HIV-1-infected MDDC with saturating concentrations of rCD85j or non-conjugated anti-HLA Class I mAbs and measured residual exposure of HLA Class I molecules and rCD85j ligands, respectively. After blockade with rCD85j, staining by the anti-HLA Class I-PE Ab was almost completely abolished in both uninfected and HIV-1-infected MDDC (Fig. 4b, B,E panels). In contrast, when HLA class I molecules were masked with anti-HLA Class I Abs, a fraction of HIV-1 infected MDDC was still stained by rCD85j (Fig 4b, F), whereas only a residual staining was detected on uninfected MDDC (Fig 6b, C). These results suggest that CD85j ligands distinct from HLA class I molecules are preferentially expressed on HIV-1-infected MDDC.

## Discussion

In this study, we examined changes in the NK cell repertoire and functions occurring in response to early interaction with HIV-infected DC, using an autologous *in vitro* NK/MDDC coculture system. We found that NK cell receptor expression is modulated in response to HIV-1 infection of MDDC, including strong CD85j down-regulation. Remarkably, the CD85j^+^ NK cell subset effectively inhibited HIV-1 replication in MDDC by affecting a post-entry step of viral cycle. CD85j^+^ NK cell-control of HIV-1 replication was apparently independent of NK cell cytotoxicity or release of soluble anti-viral factors, required NK-DC contact and might involve CD85j^+^ interaction with non HLA class I ligands.

CD85j, also known as LIR-1/ILT2/LILRB1, is an inhibitory receptor expressed on NK cells and other hematopoietic cells [25]. The known ligands for CD85j are classical and nonclassical HLA Class I molecules [25] and a viral ligand, UL18, encoded by human CMV [26]. The down-regulation of the CD85j receptor on NK cells after contact with HIV-1-infected MDDC observed in this study suggests receptor engagement with ligands on the MDDC surface. Thus, it is possible that inhibitory signals through the CD85j receptor may explain the loss of CD85j^+^ NK cell cytotoxicity against MDDC after coculture with HIV-1-infected MDDC (Fig. 3b). Interestingly, despite the loss of their lytic activity induced by HIV-1-infected MDDC, CD85j^+^ NK cells were far more effective than the CD85j^−^ subset in suppressing HIV-1 replication in infected MDDC. Indeed, whereas CD85j^−^ NK cells induced partial and transient viral inhibition, CD85j^+^ NK cells potently suppressed HIV-1 replication when added early to infected MDDC as well as 5 days after coculture with infected MDDC (Fig. 4). The mechanisms underlying HIV inhibition by the two NK cell subsets appear different. The partial control of MDDC infection by CD85j^−^ NK cells, observed in single-round infections and, transiently, in productive infections, may be due to both their preserved lytic capacity (Fig. 3b) and the secretion of HIV-inhibiting factors (Fig. 5) [24]. In contrast, CD85j^+^ NK cells exhibited low lytic activity and did not secrete HIV-inhibitory factors during coculture with infected MDDC, as assessed by transwell experiments (Fig. 5). Therefore, although we cannot formally rule out the secretion of inhibitory factors by CD85j^+^ NK cells at the immunological synapses with DC, it is unlikely that HIV-1 suppression by CD85j^+^ NK cells was mediated by either cytotoxicity or soluble factors. The inhibition of HIV-1 replication by CD85j^+^ NK cells was effective since the first cycle of viral replication (Fig. 2b), affecting either post-entry preintegration steps or viral transcription.

Taken together, our results support the hypothesis that inhibition of HIV-1 replication in MDDC by CD85j^+^ NK cells is directly or indirectly triggered by signals induced by interaction of the CD85j receptor on NK cells with ligands on HIV-infected MDDC. Indeed, blockade of NK CD85j interaction with MDDC surface molecules lifted HIV inhibition to various degrees. In particular, blockade of CD85j interactions with HLA Class I molecules only slightly lifted the inhibition, whereas a recombinant CD85j molecule almost totally abolished the inhibition of viral replication in MDDC. Inhibition of other NK receptors recognizing HLA class I molecules might also contribute to the partial removal of HIV-1 suppression by the anti-HLA Abs. Conversely, removal of CD85j^+^ NK cell-mediated viral inhibition by rCD85j points to the existence of a CD85j ligand other than HLA Class I A, B, C, E and G molecules, and suggests that NK cells use this ligand to inhibit HIV replication in MDDC. The results of competition experiments with anti-HLA class I Abs and rCD85j support this hypothesis. Indeed, a significant binding of CD85j was detected on HIV-1 infected MDDC after masking HLA class I molecules. It is tempting to speculate that the MDDC non-HLA class I ligand that mediates NK cell inhibition of HIV replication in HIV-1-infected MDDC might be modulated by HIV-1 or related to HIV-1 proteins. Further work is needed to identify the non-HLA class I ligand(s) recognized by the CD85j receptor on the surface of HIV-infected MDDC.

An increased expression of CD85j on NK cells has been reported in both viremic and long term nonprogressor HIV-1-infected patients [14], [27]. While the significance of this observation is unclear, the role of the CD85j receptor in HIV-1 infection has only been analyzed and discussed with respect to its inhibitory activity on NK cytolytic activity (usually measured on a target cell line) [14], [27]. In this paper, we show evidence that CD85j^+^ NK cells control HIV-1 replication in HIV-1-infected MDDC. Recently, KIR3DS1^+^ NK cells have been shown to inhibit HIV-1 replication *in vitro* in CD4 T cells [20]. It will be interesting to assess whether CD85j^+^ NK cells can also control HIV-1 replication in CD4 T cells and macrophages.

Should the interaction between CD85j^+^ NK cells and HIV-1-infected DC prove to contribute to host defences against HIV-1 infection, it is probable that it would intervene early, when NK cell activity is increased [12] and the virus has not yet disseminated. Indeed, the suppression of HIV-1 replication by CD85j^+^ NK cells in the first viral cycle, before viral spread in DC cultures, suggests that this mechanism may contribute to the early containment of the infection. Unfortunately, data on CD85j expression by NK during early HIV infection are lacking. *Ex vivo* studies of acutely infected individuals and/or simian models should provide new insights into the role of NK cells in early innate defences against HIV infection.

## Materials And Methods

### Generation of MDDC

Peripheral blood mononuclear cells (PBMC) were prepared from blood of healthy donors by Ficoll-Paque density gradient centrifugation. PBMC were obtained through the French blood bank (Etablissement Français du Sang) in the setting of EFS-Institut Pasteur Convention. A written agreement was obtained for each donor to use the cells for clinical research according to French laws. Our study was approved by IRB, external (Etablissement Français du Sang Board) as required by French law and internal (Biomedical Research Committee Board, Institut Pasteur) as required by Pasteur Institute. CD14^+^ PBMC were isolated with anti-CD14 MicroBeads, MS+/RS+ columns and a MiniMACS separator. Monocyte-derived DC were generated from CD14^+^ PBMC (10^6^/ml) by seeding in 6-well plates in DMEM medium plus 10% FCS; rhIL-4 and rhGM-CSF were added at final concentrations of 500 and 1000 U/ml, respectively, on days 0, 2 and 4. On day 5, floating immature DC (iDC) were transferred to new plates at a concentration of 3×10^5^ cells/ml. Phenotype of iDC was verified by CD80, CD83, HLA DR and HLA Class I expression and the absence of CD14. Maturation capacity of iDC was assessed upon LPS stimulation.

### HIV-1 infection of MDDC

iDC were infected either with 10^−1^ m.o.i of the R5 HIV-1_Bal_ strain or of NL.4/nef^-^/Bal env/GFP HIV-1, containing the green fluorescent protein (GFP) gene in the place of *nef* (a gift from P. Ancuta [28]), referred to hereafter as HIV-1/GFP. For single round infections, Bal Env pseudotyped NL-Luc-E^-^R^+^ HIV-1 particles were produced and titrated as described [29] and then used at 400 ng p24/ml. After 96 h, cells were lysed with a luciferase lysis buffer (Promega, Paris, France) and the luciferase activity was measured with a Luminometer (Glomax, Turener Biosystem, Promega, Paris, France). All infections were performed by spinoculation (1 h at 800 *g* followed by 1 h incubation at 37°C).

### Isolation of NK cells

NK cells were negatively selected by using the NK cell isolation kit MicroBeads, MS+/RS+ columns and a MiniMACS separator. The percentage of CD3-CD56^+^ NK cells in the isolated population, evaluated by flow cytometry, was>95%. NK cells were frozen in 90% fetal calf serum, 10% DMSO, until cocultured with autologous MDDC. We have previously established that NK cell activity was not affected by freezing [21], [22], To purify CD85j NK cell subsets, NK cells were incubated with an antiCD85j MAb (Beckman Coulter, Paris, France) followed by anti-mouse IgG microbeads. The proportion of NK cells expressing the CD85j receptor before enrichment was 29±16%. Purified preparations contained more than 90% of CD85j^+^ cells.

### NK/MDDC coculture

NK/MDDC were cocultured in DMEM+10% FCS in 24-well plates at a ratio of 1∶5. An NK-DC ratio of 1∶5 that induced optimal activation and proliferation of NK cells in preliminary studies was chosen for experiments. After 5 days of culture with HIV-infected or uninfected MDDC, NK cells were collected and analysed. Cell viability during cocultures was measured by propidium iodine (PI) staining: percentages of PI stained MDDC at day 14 of coculture were 25%±11 with total NK cells or CD85j^−^ NK cells and 13±6 with CD85J^+^ NK cells. PI staining of NK cells was always below 5%. In Transwell experiments, NK cells were cocultured with uninfected or HIV-1_Bal_-infected MDDC in the bottom of the wells. HIV/GFP-infected MDDC were added to the inserts and GFP expression was recorded over time.

### Proliferation and phenotypic analysis

NK cells (5×10^4^) were incubated with MDDC (2.5×10^5^) for 5 days in DMEM plus 10% FCS in 96-well round-bottom microtiter plates. NK were loaded with 5 µM CFSE before culture and proliferation was analyzed by measuring the decrease in fluorescence intensity, simultaneously with NK receptor expression. For the detection of NK cell receptors by flow cytometry, MAbs from Beckman Coulter and RD Systems were used (Paris, France).

### Analysis of NK cell cytolytic activity against HIV-infected MDDC

This study was based on immunostaining of NK cells (CD3-CD8+/-CD16+CD56+) and detection of target MDDC cell lysis by caspase flow cytometry (CyToxiLux kit, Oncoimmunin Inc, USA). Specific lysis of HIV-infected MDDC populations was evaluated by combining caspase detection with MDDC infection by an HIV-1/GFP virus. Autologous MDDC were infected with HIV1/GFP seven days before the test, then surface-stained (following the manufacturer's instructions) and placed in contact with NK cells from 5-day MDDC cocultures for one hour in the presence of a fluorogenic caspase substrate. After incubation, lysis was analysed by flow cytometry gating on target cells (surface staining and GFP expression). Measures of NK cytolytic activity by caspase assay and the chromium release technique showed good correlation (r = 0.89)

#### Blocking experiments

Blocking experiments were performed with optimal concentrations of either anti-HLA class I antibodies or recombinant CD85j protein. MDDC were incubated with 10 µg/ml anti-HLA-class I mAb W6/32 (Serotec, Paris, France), or anti-HLA-class G mAb (Serotec, Paris, France), or anti-HLA-class E Mab (Serotec, Paris, France) or a mix of the three mAbs, or with a recombinant Fc-CD85j (rCD85j) fusion protein (RD System, Paris, France) at 20 µg/ml for 30 min at room temperature before adding NK cells. Antibodies or rCD85j were added to the cocultures every two days.

#### Statistical analyses

All experiments were done in independent triplicates or quadruplicates. Data were analyzed with nonparametric (Mann-Whitney test) and parametric tests (Student's two-sided paired *t*-test). The results are expressed as means±SE.
